# Protocol for model-guided alternating high-temperature thermotherapy to eliminate major viruses from diverse sugarcane cultivars

**DOI:** 10.1016/j.xpro.2026.104808

**Published:** 2026-08-27

**Authors:** Guo-Qiang Huang, Jin-Long Guo, Feng Li, Qian Dong, Tung-Yu Hsieh

**Affiliations:** 1 National Engineering Research Center of Sugarcane, Fujian Agriculture and Forestry University, Fuzhou, China; 2 Wuyou Ecological Agriculture Co., Ltd., Fuqing, China; 3 Fujian Tianyuan Shiguang Leisure Agriculture Co., Ltd., Fuqing, China; 4 Lanxi Agricultural Technology Co., Ltd., Fuqing, China; 5 Cai Die Biological Technology Co., Ltd., Yongtai, China; 6 Fujian Universities and Colleges Engineering Research Center of Modern Facility Agriculture, Fujian Polytechnic Normal University, Fuqing, China; 7 School of Food and Biological Engineering, Fujian Polytechnic Normal University, Fuqing, China; 8 Key Laboratory of Genetics, Breeding and Multiple Utilization of Crops, Ministry of Education, Fujian Agriculture and Forestry University, Fuzhou, China; 9 Key Laboratory of Biological Breeding for Fujian and Taiwan Crops, Ministry of Agriculture and Rural Affairs, Fujian Agriculture and Forestry University, Fuzhou, China

**Keywords:** Microbiology, Plant sciences, Molecular Biology, Biotechnology and bioengineering

## Abstract

Vegetatively propagated sugarcane can accumulate multiple viruses that compromise planting-material health. Here, we present a model-guided alternating high-temperature thermotherapy (AHTT) protocol to eliminate major viruses from diverse sugarcane cultivars. We describe preparation and disinfection of single-bud axillary-bud cuttings, planting, thermal preconditioning, and a 12-day AHTT regimen. We then detail post-thermotherapy recovery, health scoring, leaf sampling, total-RNA extraction, control-validated one-step RT-PCR, result interpretation, and the detection of additional sugarcane viruses.

For complete details on the use and execution of this protocol, please refer to Huang et al*.*^1^

## Highlights

• Steps to prepare and disinfect single-bud sugarcane axillary-bud cuttings
• Details to recover shoots and score post-thermotherapy health on a 0–3 scale
• Guidance to confirm virus elimination by control-validated one-step RT-PCR

## Abstract (editor highlights)

Publisher’s note: Undertaking any experimental protocol requires adherence to local institutional guidelines for laboratory safety and ethics.

## Graphical abstract

**Figure:**
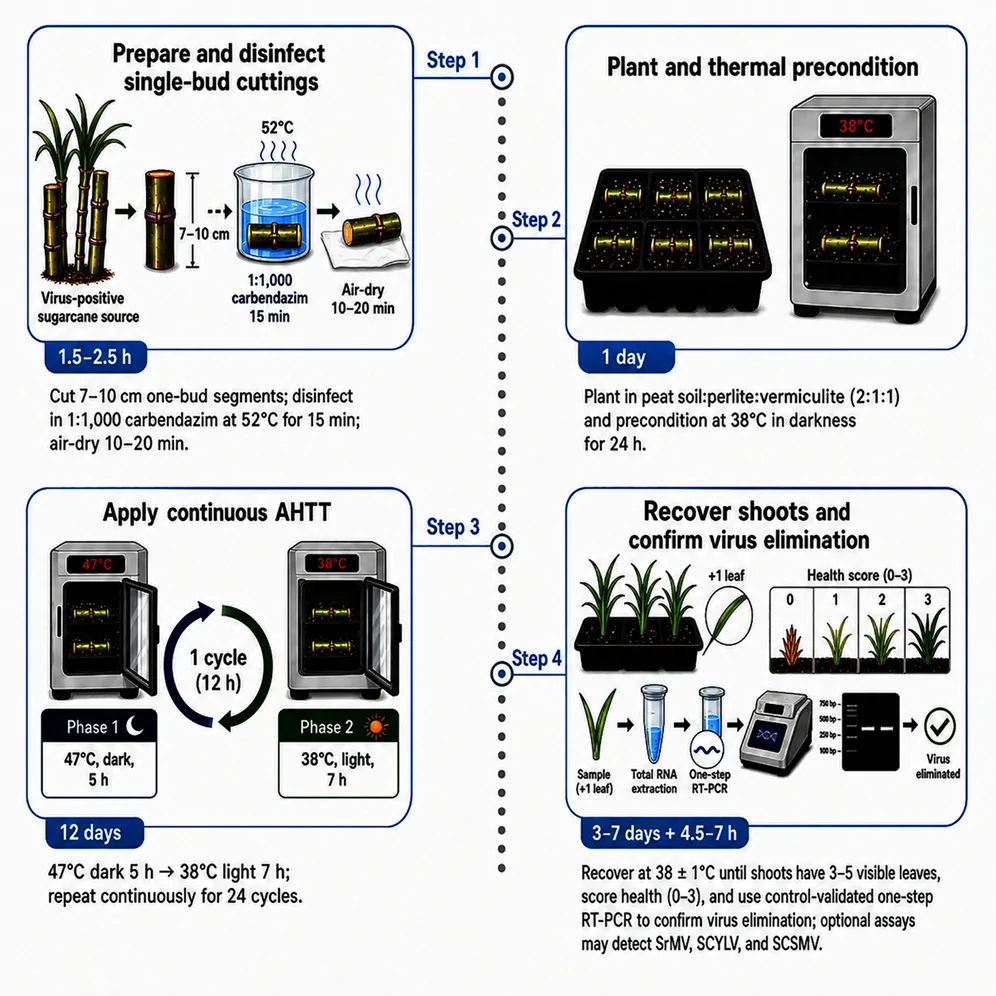

## Before you begin

### Innovation

This protocol replaces continuous heat exposure with a model-guided alternating high-temperature regimen and combines plant recovery with control-validated molecular diagnosis. It builds on established thermotherapy frameworks and the associated AHTT optimization study.^1^^,^^2^ Compared with meristem-culture-based virus elimination in sugarcane, the cutting-based workflow reduces dependence on tissue-culture expertise during the initial virus-elimination stage while retaining molecular confirmation of virus-free regenerants.^3^ The alternating design couples high-temperature dark pulses with moderate-temperature light intervals to balance viral suppression and bud recovery. The AHTT regimen was originally model-developed and experimentally optimized for SCMV-infected cv. Xuezhe. Post-publication operational validation across nine cultivars and source-positive combinations involving SrMV, SCYLV, and SCSMV expanded the workflow beyond a single genotype-virus system. Quantitative decision rules use sprouting, health index, and RT-PCR outcomes to guide cautious genotype-specific adjustment.

### Institutional permissions

This protocol uses cultivated sugarcane and naturally occurring or laboratory-maintained sugarcane viruses. It does not involve human participants, animals, endangered or protected plant species, genetically modified organisms, or regulated field release. Accordingly, no ethics or institutional review approval is required for the work described. Follow routine institutional biosafety practices and any phytosanitary, quarantine, or germplasm-transfer requirements that apply when plant or virus-positive material is maintained or moved.

### Experimental planning and diagnostic controls

**Figure 1:**
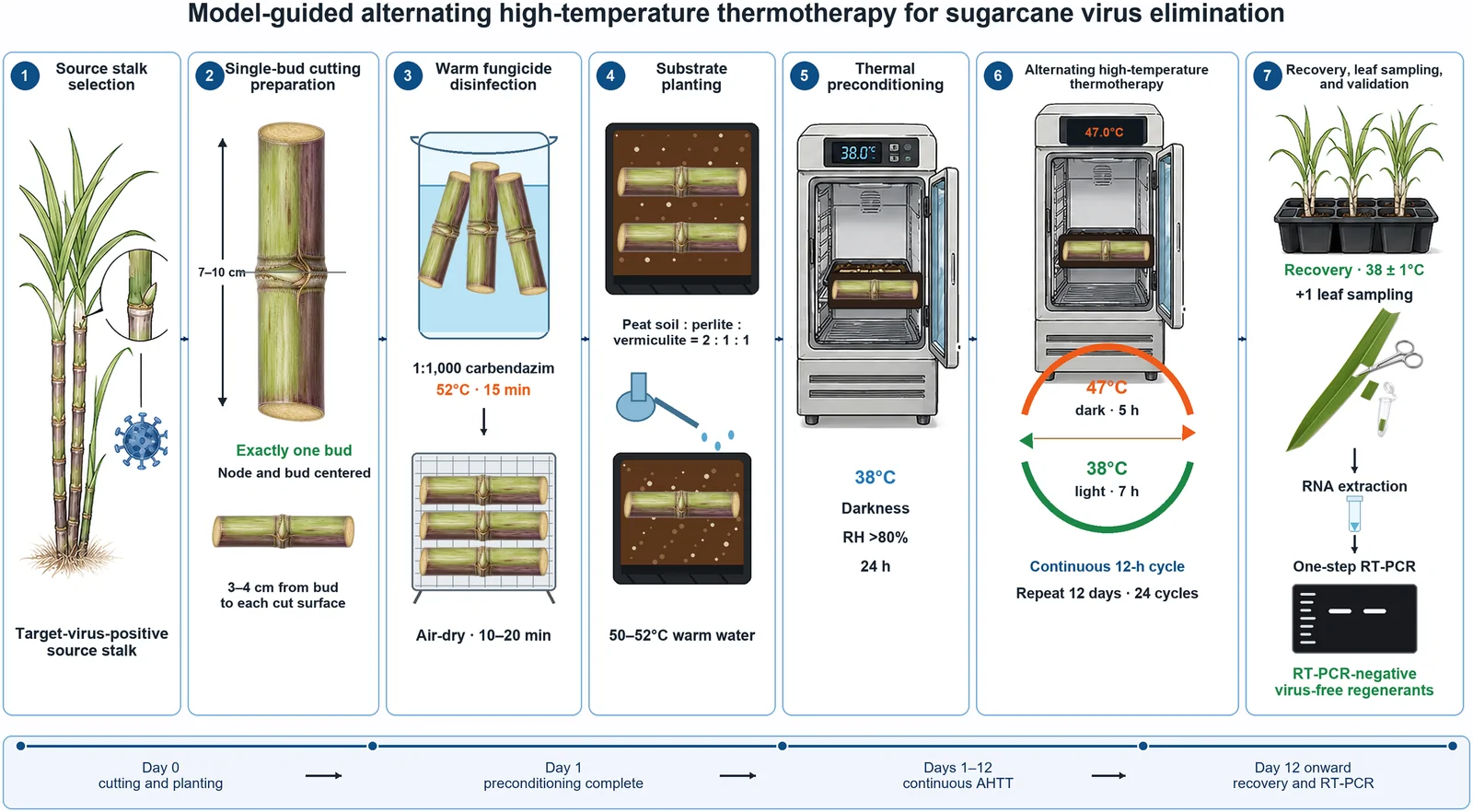
Workflow of model-guided alternating high-temperature thermotherapy for sugarcane virus elimination Target-virus-positive stalks are cut into 7–10-cm single-bud axillary-bud cuttings, disinfected in 1:1,000 carbendazim at 52°C for 15 min, planted in peat soil:perlite:vermiculite (2:1:1), and preconditioned at 38°C in darkness for 24 h. One AHTT cycle comprises 47°C for 5 h in darkness followed immediately by 38°C for 7 h under light. Repeat this continuous 12-h cycle for 24 cycles over 12 consecutive days. After AHTT, maintain the trays at 38 ± 1°C for recovery and classify shoots as virus-free only after control-validated RT-PCR. SCMV-positive cv. Xuezhe is the primary model-optimized implementation; post-publication experiments operationally validated the workflow across nine cultivars for source-positive SrMV, SCYLV, and SCSMV combinations.

Plan the biological replicates, chamber capacity, and diagnostic controls before cutting the source stalks. Figure 1 summarizes the complete workflow.

1. Confirm access to a programmable climate chamber, propagation trays, warm-water treatment equipment, RNA-extraction equipment, and one-step RT-PCR facilities.
2. Select the sugarcane genotype and target virus. For initial implementation, use SCMV-positive cv. Xuezhe and the validated default AHTT condition.^1^
3. Confirm infection in each source stalk by RT-PCR before treatment. Use validated sugarcane virus-detection assays and include positive, negative, and no-template controls.^2^
4. For SCMV, account for the substantial sequence diversity reported among South China isolates when selecting or validating a degenerate-primer assay.^3^
5. For SrMV, SCYLV, SCSMV, or another virus, validate the target-specific assay and expected product size before changing the thermal regimen, and use standard ambiguity codes when degenerate primers are required.^2^^,^^4^^,^^5^^,^^6^^,^^7^
  Table 1 summarizes the empirical AHTT adaptation guide, and Table 2 summarizes the primer sequences, expected product sizes, and cycling programs used for sugarcane virus detection.

**Table 1:** Empirical AHTT adaptation guide

Genotype or outcome	High-temperature phase	Light recovery phase	Action
Heat-sensitive genotype	46°C for 6–7 h	38°C for 5–6 h	Start cautiously and prioritize survival
Standard genotype	47°C for 5 h	38°C for 7 h	Use as the default condition
Virus persists; health index ≥1	47–48°C for 3–5 h	38°C for 7–9 h	Increase intensity in one variable at a time
Severe injury	≤46°C for ≥6 h	38°C for ≤6 h	Reduce thermal pressure

Use this guide only for cautious empirical adaptation. The continuous 47°C/38°C schedule (one 12-h cycle repeated for 24 cycles over 12 days) remains the validated default for cv. Xuezhe; change one thermal variable at a time and evaluate survival together with virus status.

**Table 2:** Primer sequences, expected product sizes, and cycling programs for sugarcane virus assays

Target	Primer sequences (5′→3′)	Product	Cycling program
SrMV	F: ACAGCAGAWGCAACRGCACAAGC R: CTCWCCGACATTCCCATCCAAGCC	860 bp	45°C 30 min; 94°C 2 min; 35 × (94°C 30 s, 55°C 1 min, 72°C 1 min); 72°C 5 min
SCYLV	F: CCGCTCACGAAGGAATGTCA R: GGAGCGTCGCCTACCTATT	588 bp	45°C 30 min; 94°C 2 min; 35 × (94°C 30 s, 55°C 1 min, 72°C 1 min); 72°C 10 min
SCSMV	F: ACAAGGAACGCAGCCACCT R: ACTAAGCGGTCAGGCAAC	938 bp	45°C 30 min; 94°C 3 min; 35 × (94°C 30 s, 58°C 1 min, 72°C 1 min); 72°C 10 min
SCMV	F: GAAGAWGTYTTCCAYCAAKCWGGAAC R: AGCTGTGTGTCTCTCTGTATTCTC	906 bp	45°C 30 min; 94°C 3 min; 35 × (94°C 30 s, 55°C 30 s, 72°C 1 min); 72°C 10 min
eEF-1a	F: TTTCACACTTGGAGTGAAGCAGAT R: GACTTCCTTCACAATCTCATCATAA	103 bp	50°C 2 min; 95°C 10 min; 40 × (94°C 15 s, 60°C 60 s)

Primer sequences are written 5′→3′ and use standard IUPAC ambiguity symbols. Expected product sizes are 860 bp for SrMV, 588 bp for SCYLV, 938 bp for SCSMV, 906 bp for SCMV, and 103 bp for the eEF-1a internal reference.

**CRITICAL:** Do not begin genotype- or virus-specific optimization unless the positive control amplifies, the negative and no-template controls remain free of target bands, and the internal reference confirms amplifiable RNA.

### Define the AHTT operating window

6. Use a continuous AHTT cycle comprising 47°C for 5 h in darkness followed immediately by 38°C for 7 h under light as the validated default regimen for cv. Xuezhe.1 Treat this 47°C/38°C sequence as one 12-h cycle, repeat it continuously for 24 cycles over 12 consecutive days, and then maintain the trays at 38 ± 1°C for post-thermotherapy recovery (Table 1).
7. For heat-sensitive genotypes, begin at 46°C for 6–7 h in darkness and retain a 38°C light recovery phase for 5–6 h.
8. If virus remains detectable and the mean health index is ≥1, increase the high-temperature phase cautiously within 47–48°C for 3–5 h and retain a 38°C light recovery phase for 7–9 h. Do not intensify treatment when severe injury is present.
9. Monitor sprouting rate, health index, and target-virus RT-PCR results for every adjustment.

***Note:*** Model-guided refers to the quantitative experimental design and response-surface interpretation used to define an adjustable operating window, rather than a single universal heat treatment.^1^

### Prepare plant material and diagnostic controls

10. Collect fresh stalks at the elongation to early-maturity stage. Operationally, use plant cane at approximately 180–240 days after planting or ratoon cane at approximately 150–210 days after ratooning, and record both crop age and visible developmental stage.
11. Exclude over-mature, dehydrated, mechanically injured, or visibly contaminated stalks.
12. Prepare target-virus-positive RNA, virus-free sugarcane RNA, and no-template controls for every RT-PCR run.
13. Include the sugarcane eEF-1a assay or another validated internal plant reference to verify RNA quality and amplification competence.^8^

**CRITICAL:** Bud physiological status strongly influences thermotolerance and sprouting. Process fresh stalks with intact axillary buds whenever possible.

## Key resources table

**Table:** 

REAGENT or RESOURCE	SOURCE	IDENTIFIER
**Biological samples**		

Fresh sugarcane stalks	Local field or germplasm nursery	Elongation to early maturity; confirm target-virus status before AHTT
SCMV-, SrMV-, SCYLV-, or SCSMV-positive source material	Authors’ germplasm collection	Naturally infected sugarcane; maintain under applicable phytosanitary rules

**Chemicals, peptides, and recombinant proteins**		

50% carbendazim wettable powder	Commercial supplier	Prepare a 1:1,000 dilution; 0.5 g active ingredient/L
Peat soil	Commercial horticultural supplier	Sterile or pasteurized; substrate component
Perlite	Commercial horticultural supplier	Horticultural grade; substrate component
Vermiculite	Commercial horticultural supplier	Horticultural grade; substrate component
RNase-free water	Commercial supplier	Molecular-biology grade
Agarose	Commercial supplier	Molecular-biology grade
Liquid nitrogen	Local supplier	For immediate tissue freezing

**Critical commercial assays**		

TransZol Up Plus RNA Kit	TransGen Biotech	Cat# ER501; plant total-RNA extraction
TransScript One-Step RT-PCR SuperMix	TransGen Biotech	Cat# AT411 or equivalent
100-bp DNA ladder	Commercial supplier	Covers 100–1,500 bp

**Deposited data**		

Associated AHTT optimization dataset	Huang et al.^1^	https://doi.org/10.3389/fpls.2026.1752582
Operational nine-cultivar validation dataset	This protocol	Reported in Table 3; raw data available from the technical contact

**Experimental models: Organisms/strains**		

*Saccharum officinarum* cv. Xuezhe	Authors’ germplasm collection	Chewing cane
*Saccharum* spp. cultivars Huangpi 1, Qingpi 1, Guitang 42, Guangxi Baiyu, Yunnan Luohan cane, Badila, ROC22, and Taoshan	Authors’ germplasm collection	Operational validation materials

**Oligonucleotides**		

SCMV-F: 5′-GAAGAWGTYTTCCAYCAAKCWGGAAC-3′	Xu *et al.*^3^; IUPAC-normalized in this protocol	W = A/T; Y = C/T; K = G/T
SCMV-R: 5′-AGCTGTGTGTCTCTCTGTATTCTC-3′	Xu *et al.*^3^	906-bp SCMV amplicon with SCMV-F
SrMV-F: 5′-ACAGCAGAWGCAACRGCACAAGC-3′	Xie *et al.*^2^	W = A/T; R = A/G, as published
SrMV-R: 5′-CTCWCCGACATTCCCATCCAAGCC-3′	Xie *et al.*^2^	W = A/T; 860-bp SrMV amplicon
SCYLV-F: 5′-CCGCTCACGAAGGAATGTCA-3′	Authors’ validated assay	588-bp SCYLV amplicon
SCYLV-R: 5′-GGAGCGTCGCCTACCTATT-3′	Authors’ validated assay	588-bp SCYLV amplicon
SCSMV-F: 5′-ACAAGGAACGCAGCCACCT-3′	Authors’ validated assay	938-bp SCSMV amplicon
SCSMV-R: 5′-ACTAAGCGGTCAGGCAAC-3′	Authors’ validated assay	938-bp SCSMV amplicon
eEF-1a-F: 5′-TTTCACACTTGGAGTGAAGCAGAT-3′	Ling *et al.*^8^	Internal reference; GenBank: EF581011.1
eEF-1a-R: 5′-GACTTCCTTCACAATCTCATCATAA-3′	Ling *et al.*^8^	103-bp internal-reference amplicon

**Other**		

Programmable climate chamber	Jiaxing Zhongxin Experimental Instrument Co. or equivalent	RXZ-160B; verified 28–48°C, light/dark and humidity cycling
NanoDrop One spectrophotometer	Thermo Fisher Scientific	RNA concentration and purity
PCR thermal cycler	Commercial supplier	Supports one-step RT-PCR programs
UV or blue-light gel documentation system	Commercial supplier	Avoid saturated band exposure
Electrophoresis power supply and gel tank	Commercial supplier	Compatible with agarose gel electrophoresis
Bench-top centrifuge	Commercial supplier	Use rotor-appropriate × g settings
Propagation trays or boxes	Commercial horticultural supplier	Drainage and verified chamber fit
Sterile pruning shears or cutting blades	Commercial supplier	For single-bud cutting preparation

**Table 3:** Operational validation across nine sugarcane cultivars

Code	Cultivar	Cane type	Sprouting rate, % (mean ± SD)	SrMV before→after	SCYLV before→after	SCSMV before→after
A	Huangpi 1	Chewing cane	100.0 ± 0.0	+ → − (100%)	+ → − (100%)	+ → − (100%)
B	Qingpi 1	Chewing cane	100.0 ± 0.0	+ → − (100%)	+ → − (100%)	+ → − (100%)
C	Guitang 42	Sugarcane	96.7 ± 5.8	− → − (N/A)	+ → − (100%)	+ → − (100%)
D	Guangxi Baiyu	Chewing cane	100.0 ± 0.0	+ → − (100%)	+ → − (100%)	+ → − (100%)
E	Yunnan Luohan cane	Chewing cane	100.0 ± 0.0	+ → − (100%)	+ → − (100%)	+ → − (100%)
F	Xuezhe	Chewing cane	96.7 ± 5.8	+ → − (100%)	+ → − (100%)	+ → − (100%)
G	Badila	Chewing cane	100.0 ± 0.0	− → − (N/A)	+ → − (100%)	+ → − (100%)
H	ROC22	Sugarcane	100.0 ± 0.0	− → − (N/A)	+ → − (100%)	+ → − (100%)
I	Taoshan	Chewing cane	93.3 ± 5.8	+ → − (100%)	+ → − (100%)	+ → − (100%)

Data represent three independent treatment batches with 10 planned cuttings per batch. +, target-virus band present; −, target-virus band absent; N/A, elimination percentage is not applicable because the source material was negative before AHTT. Interpret every negative result only when positive, negative, no-template, and RNA-quality controls are valid.

**Primer notation:** All degenerate bases follow IUPAC-IUB nomenclature: W = A/T, Y = C/T, K = G/T, and R = A/G (Cornish-Bowden, 1985; https://doi.org/10.1093/nar/13.9.3021).

## Materials and equipment

### Plant material

Use fresh, target-virus-confirmed sugarcane stalks with intact axillary buds. Process stalks promptly to limit dehydration. For the primary validated workflow, use SCMV-positive *Saccharum officinarum* cv. Xuezhe.^1^

**CRITICAL:** Over-mature, dehydrated, or mechanically injured buds often recover poorly after high-temperature exposure.

### Planting substrate

Prepare the substrate immediately before planting.

**Table:** 

Component	Relative volume	Final specification
Peat soil	2 parts	Sterile or pasteurized
Perlite	1 part	Horticultural grade
Vermiculite	1 part	Horticultural grade
Total	4 parts	Peat soil:perlite:vermiculite = 2:1:1 (v/v/v)

Mix the substrate components by relative volume to obtain a 2:1:1 peat soil:perlite:vermiculite formulation (v/v/v).

Prepare sufficient substrate to provide a 6–8-cm base layer and to cover the cuttings lightly. Use on the day of preparation. If storage is unavoidable, keep in a clean, covered container at 20–25°C for no longer than 7 days; discard contaminated, compacted, desiccated, or waterlogged substrate.

### Carbendazim disinfection solution

**Table:** 

Component	Amount	Final concentration
50% carbendazim wettable powder	1.0 g	0.5 g active ingredient/L
Water	Bring to 1.0 L	1:1,000 dilution of commercial product

Prepare 1.0 L immediately before use. The listed concentration corresponds to a 1:1,000 dilution of the commercial 50% wettable-powder product.

Prepare on the day of use and warm to 52°C immediately before disinfection. Do not reuse the solution. Dispose of it according to institutional chemical-waste rules.

### Climate chamber requirements

Use an RXZ-160B programmable climate chamber or an equivalent unit capable of maintaining 28–48°C, 0 lx during the dark phase, approximately 6,000 lx (approximately 120 μmol m^-2^ s^-1^ under the chamber lighting used here) during the light phase, ≥80% relative humidity during preconditioning, approximately 95% relative humidity during the 47°C and 38°C AHTT phases, and a programmable repeating 12-h AHTT cycle.

Manufacturer information for the example chamber: RXZ chamber resource

**CRITICAL:** Verify temperature independently with calibrated thermometers at representative shelf positions before each experimental series.

### RNA and RT-PCR reagents

Use the TransZol Up Plus RNA Kit instructions or a validated equivalent for plant total-RNA extraction.

Use the TransScript One-Step RT-PCR SuperMix instructions or a validated equivalent for one-step RT-PCR.

### One-step RT-PCR reaction mixture (20 μL)

**Table:** 

Component	Stock concentration	Volume per reaction	Final concentration
2× reaction mix	2×	10.0 μL	1×
RNA template	Validated concentration	2.0 μL	Sample dependent
Forward primer	10 μM	0.4 μL	0.2 μM
Reverse primer	10 μM	0.4 μL	0.2 μM
Enzyme mix	Kit supplied	0.4 μL	Kit specified
RNase-free water	N/A	6.8 μL	N/A
Total	N/A	20.0 μL	N/A

Volumes are given per 20-μL reaction. Prepare a master mix with 10% excess and add RNA template separately to minimize cross-contamination.

***Note:*** Prepare a master mix for the number of reactions plus 10% excess to account for pipetting loss.

## Step-by-step method details

### Preparation of single-bud axillary-bud cuttings

**Timing: 1–2 h, depending on sample number**

Here, we describe how to prepare uniform single-bud cuttings while preserving intact buds and sufficient stalk tissue to withstand heat exposure.

1. Select fresh sugarcane stalks with intact axillary buds.
2. Remove leaf sheaths gently without touching or cutting the buds.
3. Use a sterile blade or pruning tool to cut each stalk into one-bud segments 7–10 cm long and 3–4 cm in diameter.
4. Cut at the midpoints of adjacent internodes.
  a. Center the node within each segment.
  b. Keep each cut surface at least 3–4 cm from the axillary bud.
  c. Discard segments with injured buds, cracked internodes, visible fungal growth, or severe dehydration.

**CRITICAL:** The 3–4-cm buffer reduces heat-amplified wound injury and desiccation around the bud.

**Pause point:** If immediate disinfection is impossible, wrap cuttings in moist sterile paper and hold at 20–25°C for no longer than 4 h.

### Fungicide disinfection and wound drying

**Timing: 30–45 min**

Here, we describe how to reduce surface contamination and dry cut surfaces before planting without prolonging chemical or heat exposure.

5. Prepare a 1:1,000 carbendazim solution by dispersing 1.0 g of 50% carbendazim wettable powder in water and bringing the final volume to 1.0 L.
6. Warm the solution to 52°C and verify the temperature with a calibrated thermometer.
7. Immerse the single-bud cuttings completely for 15 min while maintaining 52 ± 1°C.
8. Remove the cuttings and air-dry them on clean absorbent paper at 20–25°C until no visible surface water remains; typically 10–20 min.

**CRITICAL:** Wear gloves, laboratory coat, and eye protection. Follow institutional chemical-safety and disposal requirements.

### Planting of disinfected cuttings

**Timing: 30–60 min**

Here, we describe how to plant disinfected cuttings uniformly to minimize differences in aeration, moisture, and burial depth.

9. Add prepared substrate to trays or propagation boxes to a depth of 6–8 cm.
10. Place each cutting horizontally on the substrate without direct contact between adjacent cuttings.
11. Orient all buds laterally in the same direction to facilitate observation.
12. Cover the cuttings with substrate until the stalk surfaces are just covered.
13. Saturate the substrate with water at 50–52°C.
14. Allow excess water to drain for 10–15 min before chamber transfer.

**CRITICAL:** Do not bury the cuttings deeply; excessive coverage delays sprouting and increases anaerobic stress.

### Thermal preconditioning

**Timing: 24 h**

Here, we describe how to acclimate planted buds before initiating the alternating high-temperature cycle.

15. Transfer the planted cuttings to the programmed climate chamber and maintain 38°C, darkness, and >80% relative humidity for 24 h.

***Note:*** Thermal preconditioning reduces abrupt heat shock and supports subsequent bud survival.

### Alternating high-temperature thermotherapy

**Timing: 12 days**

Here, we describe how to apply the validated continuous 12-h AHTT cycle and monitor moisture and chamber performance throughout the 12-day treatment.

16. Program phase 1 at 47°C for 5 h in darkness, 0 lx, and approximately 95% relative humidity.
17. Immediately follow with phase 2 at 38°C for 7 h under approximately 6,000 lx light and approximately 95% relative humidity.
18. Repeat steps 16 and 17 continuously as one 12-h AHTT cycle for 12 consecutive days (24 cycles in total).
  a. Treat one complete cycle as 47°C for 5 h in darkness followed immediately by 38°C for 7 h under light.
  b. Do not insert an intervening lower-temperature holding period during the 12-day AHTT treatment.
19. After buds emerge, irrigate the substrate thoroughly with 50–52°C water every 3 days.
  a. Prevent substrate drying without causing waterlogging.
  b. Record chamber temperature, humidity, and light for every 12-h cycle.

**CRITICAL:** Do not permit substrate drying. Heat and water deficit act synergistically and can cause irreversible bud injury.

***Note:*** Use the following decision guide only for empirical adaptation; the continuous 47°C/38°C cycle remains the validated default for cv. Xuezhe.1

### Post-thermotherapy recovery and health scoring

**Timing: 3–7 days, depending on genotype and growth conditions**

Here, we describe how to recover shoots under moderate conditions and quantify post-thermotherapy injury before molecular sampling.

**Figure 2:**
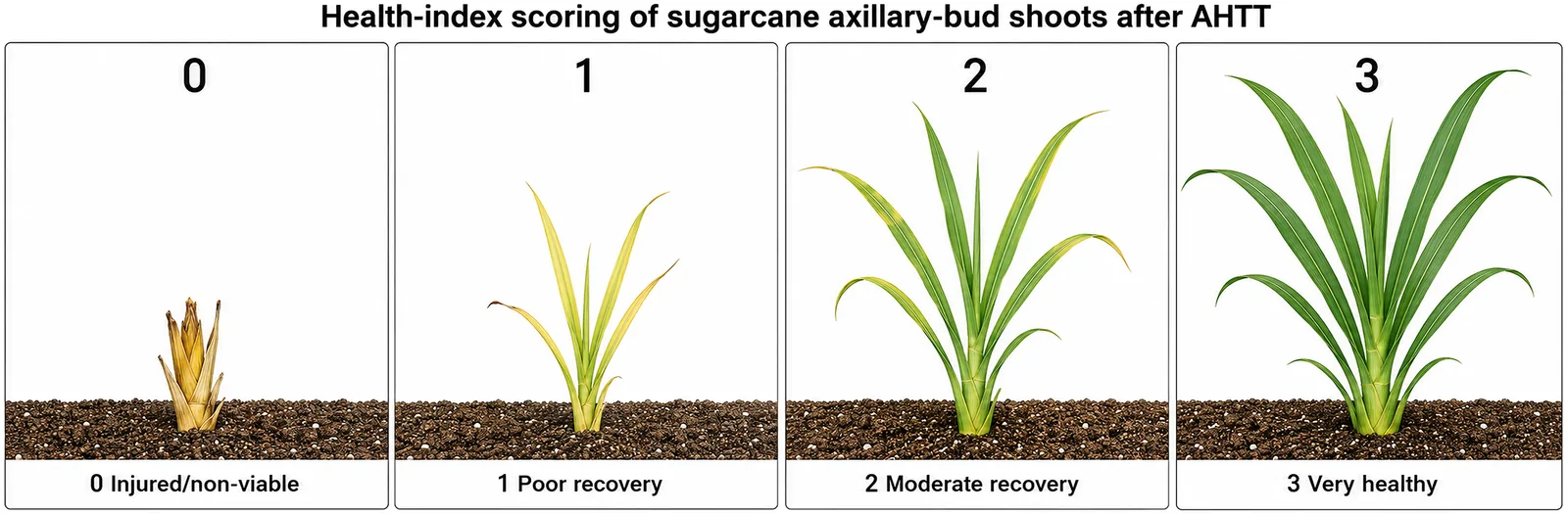
Health-index scoring system for sugarcane axillary-bud shoots after AHTT Scores range from 0 to 3: 0, injured or non-viable buds without expanded true leaves; 1, predominantly yellow weak shoots; 2, mostly green shoots with slight yellowing; and 3, vigorous shoots with fully expanded green leaves and normal growth. The formal classification follows the qualitative criteria used in the associated study.^1^

20. Stop the AHTT program after 24 cycles (12 days) and maintain trays at 38 ± 1°C, 70–85% relative humidity, a 12-h light/12-h dark photoperiod, and 100–150 μmol m^-2^ s^-1^ photosynthetic photon flux density.
21. Maintain the substrate evenly moist without waterlogging until shoots develop 3–5 visible leaves.
22. Record sprouting and score each shoot on a 0–3 scale.
  a. Score 0 for injured or non-viable shoots.
  b. Score 1 for poor recovery.
  c. Score 2 for moderate recovery.
  d. Score 3 for very healthy shoots (Figure 2).

**CRITICAL:** Treat sprouting below 70% or a mean health index below 1 as evidence of excessive thermal intensity.

### Leaf sampling and total-RNA extraction

**Timing: 1.5–3 h**

Here, we describe how to collect actively growing leaf tissue and preserve RNA integrity for diagnostic testing.

23. Collect the +1 leaf, defined here as the youngest fully expanded leaf, from each recovered shoot with 3–5 visible leaves.
24. Freeze each sample immediately in liquid nitrogen or begin extraction within 5 min.
25. Label each sample with genotype, treatment, biological replicate, and plant number.
26. Extract total RNA with the TransZol Up Plus RNA Kit or an equivalent plant RNA kit.
  a. Follow the manufacturer’s instructions.
  b. Use the recommended procedures for polysaccharide- and phenolic-rich tissue.
27. Measure RNA concentration and A260/A280 purity with a NanoDrop One or equivalent instrument. Use RNA with A260/A280 approximately 1.8–2.1 and a concentration validated for the selected kit.
28. Use the RNA immediately or store it at −80°C. Avoid more than one freeze–thaw cycle before diagnostic RT-PCR.

**CRITICAL:** Weak amplification in both virus and internal-reference reactions indicates poor RNA quality or inhibition; do not interpret the virus result.

### One-step RT-PCR detection and interpretation

**Timing: 3–4 h**

Here, we describe how to detect SCMV and, when needed, additional sugarcane viruses using IUPAC-normalized primers and explicit assay controls (Table 2).

29. Prepare each 20-μL one-step RT-PCR reaction on ice using the assay framework described by Xie et al. (2009).^2^
  a. For SCMV, add SCMV-F (5′-GAAGAWGTYTTCCAYCAAKCWGGAAC-3′).
  b. Add SCMV-R (5′-AGCTGTGTGTCTCTCTGTATTCTC-3′).
  c. Use the primer pair to amplify the expected 906-bp product.5

***Note:*** Xu *et al.* originally reported the SCMV-F5 ambiguity positions using nonstandard X and F symbols; the same primer is designated SCMV-F in this protocol. The sequence is standardized here to IUPAC-IUB nomenclature: W = A/T, Y = C/T, and K = G/T.^3^^,^^6^ The SrMV primers are reproduced as published by Xie *et al.* and already use the standard IUPAC symbols W = A/T and R = A/G.^2^

30. Run the SCMV program.

***Note:*** Use reverse transcription at 45°C for 30 min; initial denaturation at 94°C for 3 min; 35 cycles of 94°C for 30 s, 55°C for 30 s, and 72°C for 1 min; final extension at 72°C for 10 min; and a 4°C hold for no longer than 24 h.

31. Resolve products on a 1% agarose gel at 5–8 V cm^-1^ until the tracking dye migrates 60–70% of the gel length, typically 35–50 min, and acquire a nonsaturated image.
32. Interpret the SCMV assay only when all controls are valid.
  a. Identify an SCMV-positive sample by a clear 906-bp band.
  b. Classify a sample as SCMV-negative only when the target band is absent, the positive control amplifies, the negative and no-template controls lack the target band, and eEF-1a or another validated internal reference amplifies in every RNA-containing lane, including the negative control (Figure 3).

**Figure 3:**
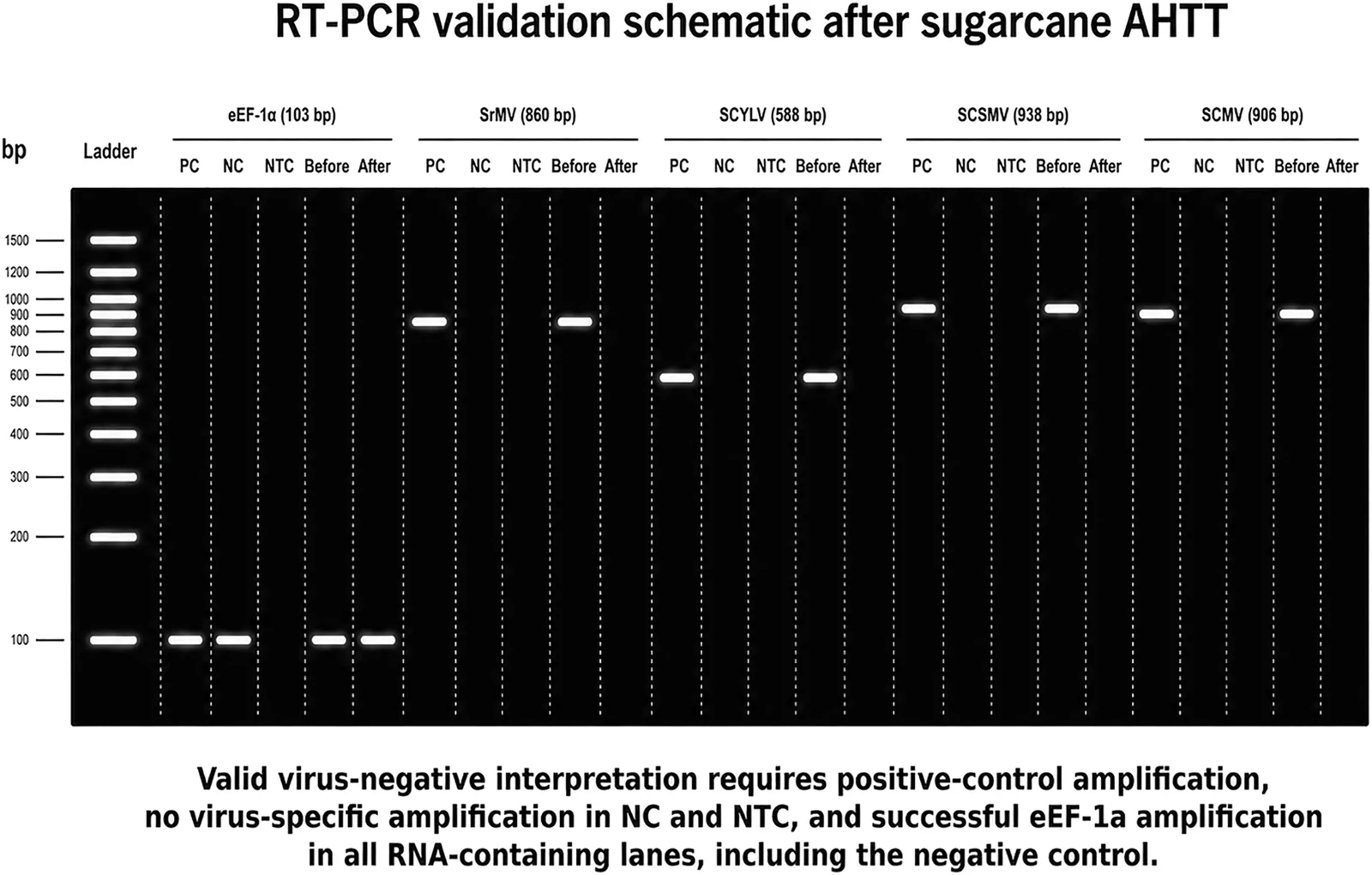
Schematic interpretation of RT-PCR controls and virus-detection outcomes after AHTT This schematic is not a raw gel image. The eEF-1a internal-reference band is expected in every RNA-containing lane with amplifiable template, including the positive control, the healthy negative control, the pretreatment sample, and the post-treatment sample, but not in the no-template control. For each virus assay, the positive control and virus-positive pretreatment sample show the expected target band, whereas the negative control, no-template control, and successfully treated post-AHTT sample lack that virus-specific band. Virus-negative status requires all controls to be valid. PC, positive control; NC, negative control; NTC, no-template control.
33. For high-value germplasm, repeat RT-PCR on a newly collected +1 leaf before large-scale propagation.

### Optional detection of additional sugarcane viruses

Use the following assays only after validating primer specificity, analytical sensitivity, and controls under the local instrument and reagent conditions.^2^^,^^7^^,^^8^

**CRITICAL:** The eEF-1a assay is an internal RNA-quality and amplification control, not a virus-detection assay.

## Expected outcomes

The validated continuous AHTT regimen (47°C for 5 h in darkness followed by 38°C for 7 h under light as one 12-h cycle, repeated for 24 cycles over 12 consecutive days) followed by recovery at 38 ± 1°C should produce viable regenerated shoots and control-validated virus-negative RT-PCR results. In cv. Xuezhe, three independent batches of 10 cuttings produced 97% sprouting, a mean health index of approximately 2, and 100% SCMV elimination among regenerated shoots.1 The corresponding overall RT-PCR-negative regeneration rate was approximately 97%; treat this value as a cultivar-specific reference, not a universal performance claim.

Post-publication operational validation extended the same continuous 12-h AHTT workflow to nine sugarcane cultivars in three independent 10-cutting batches per cultivar. All source-positive genotype-virus combinations tested for SrMV, SCYLV, and SCSMV were RT-PCR-negative after AHTT when assay controls were valid (Table 3). Because Guitang 42, Badila, and ROC22 were SrMV-negative before treatment, SrMV elimination is not calculable for those cultivars and is reported as N/A rather than 0%. These data support transferability but do not replace genotype-specific optimization or independent diagnostic validation.

## Quantification and statistical analysis

Calculate sprouting rate (%) = 100 × number of sprouted cuttings/number of planted cuttings.

Calculate virus elimination among regenerants (%) = 100 × number of RT-PCR-negative regenerants/number of regenerated plants tested. Calculate this percentage only for source-positive genotype–virus combinations.

Calculate overall protocol success (%) = sprouting rate × virus elimination among regenerants/100.

Use at least three independent biological replicates when adapting the protocol. Report the number of cuttings, mean ± SD (or mean ± SEM, explicitly identified) for continuous or rate outcomes, and the positive/negative counts for every genotype–virus combination.

### Optional downstream shoot-tip culture or micropropagation

Use recovered, repeatedly RT-PCR-negative shoots as starting material for established sugarcane shoot-tip culture or micropropagation when clonal multiplication is required.^9^ This optional downstream step is not required for validation of AHTT efficacy and should be documented separately.

## Limitations

This protocol requires a programmable chamber with accurate temperature, humidity, light, and cycle control. Chamber-position effects and substrate moisture can cause uneven survival.

Thermal tolerance varies among sugarcane genotypes. Adjust only one thermal variable at a time, and interpret virus elimination together with sprouting and health index.^10^

RT-PCR detects viral RNA only above the assay’s detection threshold. Poor sampling, degraded RNA, or failed controls can produce false-negative results. Repeat testing or use a complementary assay for high-value germplasm.^2^^,^^8^

The operational nine-cultivar results extend the evidence base but remain unpublished validation data. They should not be interpreted as proof that the validated regimen transfers unchanged to every sugarcane genotype, virus strain, or infection titer.

AHTT is a front-end sanitation step for producing improved explant or planting material; it is not by itself a complete certification or large-scale healthy-seedling production system.

## Troubleshooting

### Problem 1

**Problem:** Low sprouting rate after AHTT.

Relevant step(s): Steps 1–22.

### Potential solution

Use fresher stalks, verify substrate moisture and shelf temperature, and reduce the high-temperature duration for the next independent batch.

### Problem 2

**Problem:** Mean health index below 1.

Relevant step(s): Steps 16–22.

### Potential solution

Lower the phase-1 temperature or duration and extend the 38°C light recovery phase. Do not intensify treatment.

### Problem 3

**Problem:** Severe yellowing during or after AHTT.

Relevant step(s): Steps 16–22.

### Potential solution

Confirm high humidity and uniform moisture, prevent direct dripping, and reduce combined heat and water stress.

### Problem 4

**Problem:** Fungal growth on cuttings or substrate.

Relevant step(s): Steps 5–14 and 19.

### Potential solution

Verify carbendazim concentration and 52°C exposure, use clean trays and sterile or pasteurized substrate, and discard contaminated cuttings.

### Problem 5

**Problem:** No amplification in virus and eEF-1a reactions.

Relevant step(s): Steps 23–32.

### Potential solution

Re-extract RNA, reduce inhibitor carryover, confirm reagent storage, and test a validated positive RNA control.

### Problem 6

**Problem:** A false-negative virus result is suspected.

Relevant step(s): Steps 23–32.

### Potential solution

Resample the +1 leaf, repeat RNA extraction and RT-PCR, and require valid positive, negative, no-template, and internal-reference controls.

### Problem 7

**Problem:** The SCMV band persists after treatment.

Relevant step(s): Steps 16–19 and 29–32.

### Potential solution

If mean health index is ≥1, increase only one thermal variable cautiously in the next independent batch. Retain the original data and control records.

### Problem 8

**Problem:** Results vary across positions within one tray or chamber.

Relevant step(s): Steps 9–19.

### Potential solution

Map shelf temperatures, standardize substrate depth and spacing, and rotate trays only if the planned design allows rotation.

### Problem 9

**Problem:** Excessive condensation or dripping occurs.

Relevant step(s): Steps 15–22.

### Potential solution

Improve drainage and airflow, prevent direct condensate contact with buds, and verify the chamber humidity sensor.

### Problem 10

**Problem:** A source-negative virus produces an elimination-rate error.

Relevant step(s): expected outcomes, Table 3.

### Potential solution

Report N/A because elimination is undefined when the pretreatment denominator is zero; do not report 0% or 100%.

## Resource availability

### Lead contact

Further information and requests for resources should be directed to Tung-Yu Hsieh (dyxie@sibs.ac.cn).

### Technical contact

Technical questions about protocol execution should be directed to Qian Dong (dongqian@fpnu.edu.cn).

### Materials availability

This protocol does not generate unique restricted reagents. Sugarcane germplasm and virus-positive or virus-free plant material are subject to institutional availability, phytosanitary restrictions, and germplasm-management rules.

### Data and code availability

The associated model-development and cv. Xuezhe validation data are described in Huang et al.^1^ The operational nine-cultivar validation data are summarized in Table 3, and the underlying records and original gel images are available from the lead contact on reasonable request. This protocol does not require custom code.

## Acknowledgments

We thank the National Engineering Research Center of Sugarcane, Fujian Agriculture and Forestry University, for sugarcane plant materials and technical support, and the participating institutions for climate-chamber, plant-recovery, and virus-detection support. The affiliated companies provided access to part of the experimental facilities, routine care of plant materials, and partial-salary support for some researchers. As corporate entities, they had no role in study design, experimental treatments or measurements, data collection or analysis, interpretation, manuscript preparation, or the decision to submit the work for publication. This research was funded by the Foundation for Science and Technology Innovation of Fujian Agriculture and Forestry University, China (grant nos. KFB23185 and KFB24030) and the 10.13039/501100012166National Key R&D Program of China (grant no. 2022YFD2301100).

## Author contributions

G.-Q.H. and J.-L.G. performed plant-material preparation, thermotherapy, recovery, and virus-detection experiments. F.L. contributed to protocol optimization, agricultural application assessment, and facility coordination. Q.D. contributed to experimental organization, molecular-detection workflow development, data interpretation, and manuscript revision. T.-Y.H. conceived and supervised the study, integrated the model-guided AHTT strategy, interpreted physiological and diagnostic outcomes, and wrote and revised the manuscript. All authors reviewed and approved the manuscript.

## Declaration of interests

F.L. and T.-Y.H. are employed by Lanxi Agricultural Technology Co., Ltd.; Wuyou Ecological Agriculture Co., Ltd.; Cai Die Biological Technology Co., Ltd.; and Fujian Tianyuan Shiguang Leisure Agriculture Co., Ltd. The affiliated companies provided access to part of the experimental facilities, routine care of plant materials, and partial-salary support for some researchers. As corporate entities, they had no role in study design, experimental treatments or measurements, data collection or analysis, interpretation, manuscript preparation, or the decision to submit the work for publication. Q.D., G-Q.H., J.-L.G., Wensen Zhang, and Zhi Cao are named inventors on Chinese invention patent ZL202310850360.X, titled “Method for eliminating sugarcane mosaic virus from Xuezhe sugarcane and producing virus-free seedlings,” granted on June 6, 2025.

## Declaration of generative AI and AI-assisted technologies in the writing process

During preparation of this work, the authors used ChatGPT Plus to support English-language editing, consistency checking, and document formatting. The authors reviewed and edited all content and take full responsibility for the content of the publication. No generative AI was used to create or alter primary research images or data; the submitted explanatory figures were drawn programmatically and verified by the authors.
